# Effects of provider incentives on dental X-raying in NHS Scotland: what happens if patients switch providers?

**DOI:** 10.1007/s10198-021-01348-3

**Published:** 2021-07-13

**Authors:** Olivier Kalmus, Martin Chalkley, Stefan Listl

**Affiliations:** 1 Section for Translational Health Economics, Department of Conservative Dentistry, University Hospital Heidelberg, Heidelberg, Germany; 2 Centre for Health Economics, University of York, York, UK; 3 Department of Dentistry - Quality and Safety of Oral Health Care, Radboudumc - Radboud Institute of Health Sciences, Nijmegen, Netherlands

**Keywords:** Financial incentives, Patient safety, Provider remuneration, Health care quality improvement

## Abstract

**Background:**

In many market settings individuals are encouraged to switch health care providers as a means of ensuring more competition. Switching may have a potentially undesirable side effect of increasing unnecessary treatment. Focusing on the most common source of medical radiation (dental X-rays), the purpose of this study was to assess whether, upon switching dentist, X-ray exposure increases depending on the type of provider payment.

**Methods:**

The analysis used longitudinal data from 2005 to 2016 covering a 5% random sample of the Scottish adult population covered by the National Health Service (NHS). Multiple fixed-effects panel regression analyses were employed to determine the correlation of provider remuneration with patients’ likelihood of receiving an X-ray upon switching to a new dentist other things equal. A broad set of covariates including a patient’s copayment status was controlled for.

**Results:**

Upon switching to a dentist who was paid fee-for-service, patients had a by 9.6%-points (95% CI 7.4–11.8%) higher probability of receiving an X-ray, compared to switching to a salaried dentist. Results were robust when accounting for patient exemption status, as well as unobserved patient and dentist characteristics.

**Conclusions:**

In comparison to staying with the same dentist, patients may be exposed to substantially more X-rays upon switching to a dentist who is paid fee-for-service. There may need to be better guidance and regulation to protect the health of those who have to switch provider due to moving and greater caution in advocating voluntary switching.

**Supplementary Information:**

The online version contains supplementary material available at 10.1007/s10198-021-01348-3.

## Background

Choice of health care provider is often dictated by location. People who move over large distances have to switch provider. In addition health care systems have increasingly seen the introduction of market mechanisms [1, 2] in which switching of providers is encouraged [3] as a means of improving competition. Contact with a new patient is an opportunity to offer treatment and so a potentially undesirable medical side effects of patient switching is excessive treatment. It is well-established that some types of provider remuneration such as fee-for-service [4], increase the propensity to treat leading for calls for it to be replaced or regulated. Where a treatment is potentially harmful this implies that there might be a risk to a patient who is either forced to or chooses to switch provider. To this end, we examine whether patients are exposed to additional ionizing radiation upon switching between dentists under different provider payment schemes. We exploit large-volume individual-level administrative data and the unique characteristics of the most frequently applied type of medical imaging: dental X-rays. We focus on variations in the use of dental radiographs when patients switch between providers who receive either fee-for-service or salary payments.

Medical radiography is associated with a radiation risk for patients. While X-rays serve a crucial role in diagnostics, radiation exposure is a well-known human carcinogen [5]. Dental X-rays are among the most used medical radiographs and the most common source of artificial radiation exposure [6]. Current clinical guidelines in many countries confirm that dental radiographs should only be used if the patient’s benefits exceed the risks that is only upon strict clinical indication [7]. Nonetheless, the procedure is frequently delivered as part of a routine examination, especially when visiting a dentist for the first time. There is little evidence on the exact carcinogenic effects of low dose radiation, such as caused by dental radiographs, because the sample sizes required to study them would need to be extremely large [8]. The consensus held by radiological protection organizations is based on the so-called linear no threshold (LNT) model, which postulates that risk of radiation induced cancer increases linearly with exposure and that there is no safe threshold [9]. Indeed, a number of studies have been able to show an association between dental X-rays and the risk for meningiomas and salivary tumors [6, 10, 11].

It has been established that health care providers adapt their behavior to financial incentives [12]. Ideally, financial rewards can be used to improve providers’ performance and the quality of services provided [13]. Empirical evidence, however, remains weak that these goals are easily achieved [14, 15]. Previous studies have found that the introduction of financial incentives has increased treatment intensity and created supplier induced demand. Evidence indicates that this often constitutes ineffective care with little effects on patient health, rather than a reduction of existing rationing [16].

Most of the existing literature on the topic has focused on general medicine, however, there is a growing body of evidence looking at dental care in particular [17, 18]. Studies show that financial incentives have the potential to increase utilization of dental check-ups [19] and increase the number of individuals under dentist supervision without a reduction in service quality [20]. However, remuneration schemes used for dentists can enable the provision of additional care with limited or no marginal benefits for patients [21] with potential adverse effects for patients [22]. Notably, fee-for-service payments have been shown to increase the probability of dental radiographs being provided to patients [23]. Ours is the first study to focus on the impact of switching dentist on this potentially harmful treatment.

Based on existing studies of the impact of fee-for-service arrangements in general and for dentistry in particular, our hypotheses were that (i) patients receive significantly more dental radiographs upon switching to another dentist; and (ii) the effect on dental radiographs upon switching to another dentist is significantly larger under fee-for-service as compared to salary payment.

## Methods

### Institutional background

Dental care in Scotland is provided through a public–private mix. Public dental care is primarily delivered by the NHS General Dental Service. For the most part GDS is provided by private dentists that have arrangements with the NHS. They are being remunerated based on a mix of capitation and fee-for-service payments. The exact arrangements of the GDS reimbursements are detailed in the Statement of Dental Remuneration [24]. There are also a number of salaried dentists that provide GDS in areas where independent private dentists did not meet local needs. Since 2014 these salaried dentists are part of the Public Dental Service (PDS), whose main responsibility is to provide GDS to patients that cannot access care from private dentists. In general patients receiving care under the GDS pay 80% of the treatment fees up to a certain limit out-of-pocket. However patients can be exempt from these charges for a number of different reasons [25]. The patient fee for a dental X-ray is independent of the dentist’s remuneration status. Private dentists are free to provide dental care to paying patients outside of the GDS. The fact that our data set does not include any information on these services should not cause problems for validity of the results. Our results would depict lower bound estimates, if X-rays are provided privately on a regular basis.

The guideline for oral health assessment published by the Scottish Dental Clinical Effectiveness Program (SDCEP) determines the situations in which dental radiographs should be taken as a diagnostic tool [26]. In particular the SDCEP determines that “routine ‘screening’ radiography (e.g., panoramic radiographs) must not be carried out and new radiographs must not be undertaken without first examining existing films or without clear justification”. In cases where radiographs are necessary, dentists should undertake all necessary steps to minimize the need for further radiography.

### Data

Our study is based on a data set originating from the GDS database, provided to us by the Information Services Division (ISD) of NHS Scotland. The data were extracted from the Management Information and Dental Accounting System (MIDAS) that contains all treatment claims made under the GDS. ISD drew a random sample of 5% all individuals registered in the GDS in 2016 and tracked them back until 2005. Our data set thus includes the full dental histories of all sampled individuals allowing us to conduct a panel analysis. In total our data set contains 4,369,961 claimed services provided to 146,239 (around 2.7% patients by 4894 different dentists over the 11-year study period. Besides the detailed information on dental claims, our data set included variables on various patient and dentist characteristics.

**Fig. 1:**
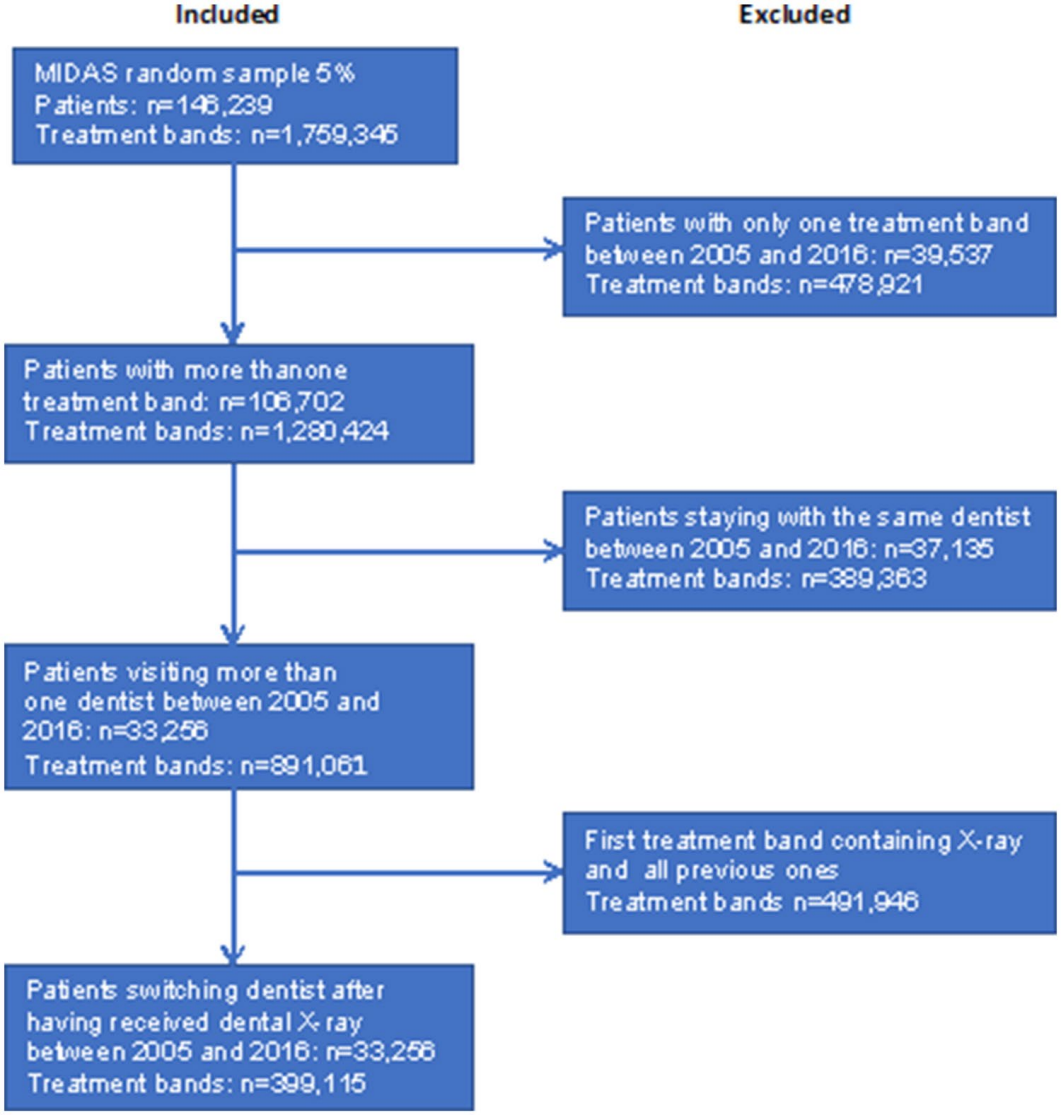
Sample selection process

We collapsed the data onto the treatment band level for analysis. Treatment bands were defined as all treatments that were initiated on the same starting date, disregarding actual date of delivery and all accompanying treatments. The reason for this choice is based on the GDS practice. When a patient visits a dentist, the latter develops a treatment plan (if necessary) that might include multiple follow-up visits. In the MIDAS database all these treatments are recorded as having the same start date. We only retained the treatment bands, where a patient visits a dentist for the first time during the study period and having received a dental X-ray during a previous treatment band by a different provider. Figure 1 illustrates this exclusion process.

### Outcomes and covariates

Table 1 summarizes the operationalization of the outcome variable and chosen covariates. The outcome variable for our analyses was the delivery of at least one dental X-ray (small dental film) to a patient during any given treatment band.

**Table 1:** Summary statistics: dependent and independent variables

*N* = 399,115	Description	Mean [Std.Dev.]
X-ray	Equals 1 if “small dental film” was claimed	28.32%
Deprivation	Deprivation quintiles of patients’ residence	
	1 (lowest deprivation)	19.75%
	2	20.23%
	3	19.81%
	4	19.98%
	5 (highest deprivation)	20.24%
Patient’s age	Patient’s age in years	40.17 [19.77]
Patient’s exemption status	Equals 1 if patient was exempt from payment for any reason	
Months since last visit	Duration since previous treatment band was started (months)	12.23 [16.03]
Dentist’s experience	Years since dentist registered with the GDS	9.84 [10.24]
Dentist’s remuneration status	Equals 1 if dentist is salaried, 0 if dentist paid fee-for-service	7.71%

The main independent variable of interest is the dentists’ remuneration status. We also take into account the dentists’ experience. This is based on the date, the dentists first registered with the GDS. While dentists theoretically could already have practiced privately beforehand, we assume that for the vast majority, this represents their professional experience accurately. The time elapsed in between treatment bands is also controlled for. We expect that the longer that duration, the higher the probability of severe changes in dental health status, which would render the provision of dental radiographs recommended in accordance with the SDECP guidelines.

Patient characteristics that we controlled for include patient age and deprivation of the area of residence. The latter is based on the Scottish Index of Multiple Deprivation that takes into account a number of socio-economic characteristics across seven different domains [27]. The scores are applied to 6976 geographical areas of equal population. Additionally, we controlled for patients being exempt from co-payments, to check to the effect of patient financial incentives. Assuming that the gender of patients and dentists is constant over time, gender is taken account of by the fixed effects in our analytical approach (see below).

### Analytical approach

This study adopts a previously established theoretical framework concerning the role of financial incentives in relation to dental X-raying [23]. The framework takes into account that X-ray provision depends on the interaction between patients and dentists. In general, the probability of a dental radiographic examination is dependent on the dentist and patient characteristics, the dentist’s remuneration scheme, the patient’s dental health status and the patient’s own financial contribution. If dentists act altruistically in the best interest of their patients, their reimbursement scheme should not be significantly associated with the probability of providing a dental radiograph.

This framework can be operationalized as a linear probability model [23]. While the binary outcome variable opens the possibility of using non-linear regression models, we chose to follow a linear approach for the practical reasons of computation speed and ease of interpretation of the regression coefficients. As we are interested in analyzing the correlation of dentist reimbursement with X-ray provision, we have to confront the fact that, relying on observational data, reimbursement status is not randomly assigned, thus creating a possible endogeneity bias. We address this issue by exploiting the longitudinal nature and panel characteristics of our data in a multiple fixed effects model. Through repeated observations of the same dentists and estimating dentist fixed effects to correct for unobserved heterogeneity, we can take into account the effect of changes in reimbursement status. Similarly, many patient characteristics are not contained in the data. To avoid biased results based on unobserved time-constant characteristics, we also estimate patient fixed effects.

We estimated the regression model for the sample of treatment bands, where patient visited a dentist for the first time and have previously received an X-ray during our study period. We are thereby able to estimate the effects of remuneration status on X-ray provision for new patients. As a control we also estimated the model for the overall sample of treatment bands. All calculations were carried out using Stata/SE 14.2 [28]; the two-way fixed effects estimations were carried out using the *felsdvreg* command.

## Results

Table 2 summarizes the descriptive statistics of our sample. Among the 399,115 treatments sessions where people visited a new dentist for the first time, 28.32% (113,010) included a dental X-ray. We observe a big difference between fee-for-service dentists 28.93% (106,567), compared to the 20.93% (6443) of salaried dentists in terms of providing a dental X-ray during the first visit.

**Table 2:** Descriptive statistics for study sample by dentist reimbursement status

*N* = 399,115	Fee-for-service dentists	Salaried dentists
X-ray (in %)	28.93%	20.94%
Deprivation (in %)		
1	19.75%	19.71%
2	20.11%	21.67%
3	19.4%	24.73%
4	19.84%	21.64%
5	20.91%	12.26%
Exempt patients (in %)	38.8%	43.89%
Mean patient age in years [SD]	40.28 [15.83]	38.8 [20.74]
Mean months since last visit [SD]	12.02 [15.83]	14.82 [18.09]
Mean dentist’s experience in years [SD]	9.92 [10.28]	8.91 [9.64]

Our two-way fixed effects regression model (Table 3) confirms the substantial difference between salaried and fee-for-service dentists observed on the descriptive level. The probability of a salaried dentist providing an X-ray for a new patient is 9.6 percentage points (95% CI [− 0.118; − 0.074]) lower than for their fee-for-service peers. We find that a patient’s deprivation (−0.0045 [− 0.0081; − 0.0011]) is negatively associated with the probability of receiving an X-ray. A patient’s exemption status (− 0.066 [− 0.073; − 0.060]) is also negatively associated with X-rays, meaning that exempt patients are less likely to receive one. A longer duration in between treatment bands increased the likelihood of the patient receiving an X-ray (0.0039 [0.0037; 0.0039]). The effect of a dentist’s experience approaches 0 in the analysis. These results were stable when adjusting for year fixed effects (Table 4).

**Table 3:** Regression results: effects on dental X-raying

*N* = 399,115	Coefficient [95% confidence interval]	Standard error (*p* value)
Dentist’s remuneration status	− 0.096 [− 0.118; − 0.074]	0.011 (< 0.000)
Deprivation	− 0.005 [− 0.008; − 0.001]	0.002 (0.01)
Patient’s age	− 0.0005 [− 0.002; 0.000]	0.001 (0.45)
Patient’s exemption status	− 0.066 [− 0.073; − 0.06]	0.003 (< 0.000)
Months since last visit	0.004 [0.0037; 0.0039]	0.00006 (< 0.000)
Dentist’s experience	− 0.0018 [− 0.0036; − 0.0000]	0.001 (0.04)

**Table 4:** Regression results: effects on dental X-raying with year fixed-effects

*N* = 399,115	Coefficient [95% confidence interval]	Standard error (*p* value)
Dentist’s remuneration status	− 0.094 [− 0.12; − 0.076]	0.013 (< 0.000)
Deprivation	− 0.004 [− 0.008; − 0.001]	0.002 (0.02)
Patient’s age	− 0.0005 [− 0.003; 0.000]	0.001 (0.53)
Patient’s exemption status	− 0.069 [− 0.076; − 0.058]	0.002 (< 0.000)
Months since last visit	0.002 [0.0027; 0.0049]	0.0003 (< 0.000)
Dentist’s experience	− 0.0018 [− 0.0036; − 0.0000]	0.001 (0.04)

## Discussion

Individuals move between health providers for a variety of reasons both non-discretionary and in response to encouragement to seek out better health care. This study shows that patients visiting a dentist for the first time are more likely to be X-rayed if the dentist is remunerated on a fee-for-service scheme, leading to potential excess radiation exposure. We focused on the association between reimbursement mechanisms and dental radiograph frequency, which allows us to identify a difference in treatment that should not exist. This difference indicates potentially inequitable treatment based on provider remuneration leading to excess radiation exposure or risk of under-treatment.

Dental radiographs are the most common form of artificial radiation exposure [6]. Clinical guidelines limit their use to situations, where the benefits outweigh the risks for the patients [26]. We confirm and add to the previous results that fee-for-service payments are associated with more dental radiographs [23] and show that the difference in frequency of X-ray provision is even more substantial during first time visits. Whilst dental radiographs are an indispensable diagnostic tool, limiting unnecessary X-rays should be among the goals of every health system. Based on the regression results presented in Table 3, and considering the care provided by salaried dentists as reference level, this would mean that switches to fee-for-service dentists during the observation period were correlated 38,315 dental X-rays in excess of switching to a salaried dentist. Accordingly, the estimated excess radiation exposure experienced by our study population, relative to if dentists were paid on a fixed salary would amount to 11,495–827,604 µSv [29].

Patients in our study sample may have moved between dentists for a variety of reasons. One of them could be that there were differences in the quality of dental services provided according to dentists’ remuneration status. Patients who chose a dentist who was paid fee-for-service may have done so because they believed this was the best dentist for them. In addition, the possibility to switch between dentists likely depends on the supply of dentists within an area. The data available for our study did not lend themselves to adjust our analyses for the quality of dental services or detailed geographic distribution of the dental workforce and this should be acknowledged as a limitation of our study.

This study has a number of implications for the regulation and oversight of dental health care provision. It illustrates a continuing risk of fee-for-service arrangements in leading to excessive X-rays and identifies an at-risk group of patients—those that have to, or choose to, switch dentist and arrive at a fee-for-service provider. It also highlights a hitherto undocumented risk of encouraging patients to switch between providers. Whilst that encouragement might be beneficial in respect of improving the market for dental health care, it might also be placing patients at risk.

## Conclusions

The provision of dental radiographs should be linked to clear benefits for the patients and not carried out as a routine treatment. Our findings indicate that patients visiting a dentist for the first time are more likely to receive a dental radiograph, if that dentist is paid fee-for-service. This highlights that fee-for-service remuneration can potentially lead to adverse effects for patients. Furthermore, these adverse effects can be exacerbated through reforms that encourage patients to shop around for providers. When implementing such reforms to improve competition, the effects of financial incentives need to be considered.

## Supplementary Information

Below is the link to the electronic supplementary material.

Supplementary file1 (PDF 487 KB)

## Data Availability

The analysis exploits individual-level claims data from NHS Scotland. Due to data protection regulations, we are not allowed to publicly share the original research data and can only provide do-files of the analysis and aggregated information about the dataset if desired.
